# A new classification of HLA-DRB1 alleles differentiates predisposing and protective alleles for autoantibody production in rheumatoid arthritis

**DOI:** 10.1186/ar2131

**Published:** 2007-03-12

**Authors:** Pierre-Antoine Gourraud, Philippe Dieudé, Jean-Frédéric Boyer, Leonor Nogueira, Anne Cambon-Thomsen, Bernard Mazières, François Cornélis, Guy Serre, Alain Cantagrel, Arnaud Constantin

**Affiliations:** 1 Service d'Epidémiologie CHU Toulouse, INSERM, U558, Université Paul Sabatier Toulouse III, Faculté de Médecine, 37 allées Jules Guesde, Toulouse Cedex 7, 31073, France; 2 Service de Rhumatologie, CHU Bichat Claude-Bernard, 46 rue Henri Huchard, Paris, 75018, France; 3 GRCB40, UFR Sciences Médicales Rangueil, 1 avenue du Professeur Jean Poulhès, Toulouse Cedex 9, 31059, France; 4 Service de Rhumatologie, CHU Toulouse Rangueil, 1 avenue du Professeur Jean Poulhès, Toulouse Cedex 9, 31059, France; 5 Laboratoire de Biologie Cellulaire et Cytologie, CHU Toulouse Purpan, Place du Docteur Baylac, Toulouse cedex 9, 31059, France; 6 GenHotel, Genopole, 2 rue Gaston Crémieux, Evry Cedex, 91057, France; 7 INSERM, U558, Faculté de Médecine, 37 allées Jules Guesde, Toulouse Cedex 7, 31073, France

## Abstract

The HLA-DRB1 gene was reported to be associated with anticitrullinated protein/peptide autoantibody (ACPA) production in rheumatoid arthritis (RA) patients. A new classification of HLA-DRB1 alleles, reshaping the shared epitope (SE) hypothesis, was recently found relevant in terms of RA susceptibility and structural severity.

We investigated the relevance of this new classification of HLA-DRB1 SE^+ ^alleles in terms of rheumatoid factor (RF) and ACPA production in a sample of French RA patients.

We studied 160 early RA patients included in a prospective longitudinal cohort of French Caucasian patients with recent-onset arthritis. RF, anticyclic citrullinated peptide 2 (anti-CCP2) and antideiminated human fibrinogen autoantibodies (AhFibA) were assessed in all patients at inclusion. The HLA-DRB1 gene was typed by PCR-sequence specific oligonucleotides probes (PCR-SSOP), and SE+ alleles were classified into four groups (S1, S2, S3P, S3D) according to the new classification.

The new classification of HLA-DRB1 SE+ alleles distinguishes predisposing and protective alleles for RF, anti-CCP2 or AhFibA production. The presence of S2 or S3P alleles is associated with both RF, anti-CCP2 or AhFibA positivity, whereas the presence of S3D or S1 alleles appears to be protective for RF, anti-CCP2 or AhFibA positivity.

The new classification of HLA-DRB1 SE^+ ^alleles is relevant in terms of autoantibody production in early RA patients by differentiating predisposing and protective alleles for RF or ACPA production.

## Introduction

Since early rheumatoid arthritis (RA) is often indistinguishable from other inflammatory joint diseases, recent-onset inflammatory synovitis poses a diagnostic and prognostic challenge to rheumatologists [1]. The identification and validation of immunologic and genetic markers with strong diagnostic and prognostic value in early RA may help rheumatologists to meet this challenge [2].

Among immunologic markers, anticitrullinated protein/peptide antibodies (ACPAs) constitute relevant tools in the diagnosis and prognosis of early RA. Citrulline is a nonstandard amino acid, generated by post-translational modifications of several proteins by deimination of arginine residues by peptidylarginine deiminases [3,4]. The citrulline moiety is the true determinant in proteins recognized by antiperinuclear factor [5], antikeratin antibodies [6], antifilaggrin antibodies [7], anticyclic citrullinated peptide (anti-CCP) antibodies [8] and anti-deiminated human fibrinogen autoantibodies (AhFibA) [9-11]. ACPAs may be detected in healthy individuals, years before the onset of symptoms of RA [12,13], and may predict progression to persistent erosive arthritis or to RA in patients with undifferentiated arthritis [14-16]. ACPAs are as sensitive as, and more specific than, rheumatoid factor (RF) for early RA diagnosis [17-19]. Furthermore, ACPAs represent a prognostic factor for erosive disease in early RA [20-24].

Among genetic markers, the HLA-DRB1 gene has been clearly involved in the pathogenesis of RA [25,26]. The association between HLA-Dw4 and RA was first reported in 1976 [27]. The development of HLA-DRB1 genotyping led to the demonstration that different HLA-DR4 alleles were not equally associated with RA and that several non-DR4 HLA–DRB1 alleles were also associated with the disease. The shared epitope (SE) hypothesis, first proposed in 1987, represents an approach to understand the molecular genetics of susceptibility to RA. The SE hypothesis assumes that HLA-DRB1 alleles encoding a highly conserved amino acid sequence, known as the SE – which is characterized by the RAA pattern at positions 72–74 of the third hypervariable region of different HLA-DRβ_1 _chains – are associated with susceptibility to RA [28]. HLA-DRB1 alleles encoding the SE were then associated with structural severity of RA [29] and have been more recently associated with production of ACPAs [9,12,24,30-32].

As was done in previous attempts [33,34], du Montcel and colleagues recently introduced a new classification of HLA-DRB1 alleles that reconsiders the SE hypothesis [35]. In terms of susceptibility to RA, this new classification suggests that the risk of developing RA depends on whether the RAA sequence occupies positions 72–74 but the risk is modulated by the amino acids at position 71 (K confers the higher risk, R an intermediate risk, A and E a lower risk) and at position 70 (Q or R confers a higher risk than D) [35-37] complexifying the classical SE epitope classification based on the presence of RAA in positions 72–74. In terms of structural severity of RA, this new classification allowed the differentiation of predisposing or protective alleles (two effects) – respectively characterized by the DRRAA or by the DERAA amino acid pattern at positions 70–74 [36] – which was not possible using the classical SE epitope classification based on the only presence of RAA in positions 72–74.

In the present study, we investigated the relevance of this new classification of HLA-DRB1 alleles in terms of RF and ACPA production in a cohort of French Caucasian patients with early RA. Interestingly, the new classification of HLA-DRB1 alleles allows the differentiation between predisposing and protective alleles for autoantibody production.

## Materials and methods

### Patients

One hundred and sixty Caucasian outpatients were selected from the Rangueil Midi-Pyrénées cohort, which involved patients with early arthritis who attended the Rangueil Hospital Department of Rheumatology between November 1992 and December 1997, according to the following criteria: the American College of Rheumatology 1987 criteria for RA [38], disease duration <1 year from the first clinical manifestation of RA, and age over 16 years.

Each individual included in the Rangueil Midi-Pyrénées cohort signed an informed consent form. The protocol was initially approved by the Committee for the Protection of Persons Participating in Biomedical Research (French law 88–1138 December 20, 1988).

### Detection of RF and ACPAs

Blood samples were collected at baseline, immediately centrifuged and stored at -80°C until assayed. RF was quantified by nephelometry according to the manufacturer's recommendations (RF Reagent, IMMAGE immunochemistry system; Beckman Coulter, Inc., Fullerton, CA, USA). Anti-CCP2 antibodies were detected by ELISA according to the instructions of the manufacturer (IMMUNOSCAN RA; Euro-Diagnostica, Arnhem, The Netherlands). AhFibA were detected with a recently developed inhouse ELISA, using *in vitro *deiminated human fibrinogen as immunosorbent [9,10]. The cut-off points of the two ELISAs were previously set so they reached the same diagnostic specificity of 98.5%.

### HLA-DRB1 genotyping and allele classification

Genomic DNA was extracted from ethylenediamine tetraacetic acid anticoagulated peripheral blood, using a standard proteinase K digestion and phenol/chloroform extraction method, in all patients at the time of inclusion. HLA-DRB1 typing and subtyping were performed by a PCR-based method, using a panel of sequence-specific oligonucleotide probes [36].

HLA-DRB1 alleles were pooled according to the new classification proposed by du Montcel and colleagues [35,36]. Briefly, the HLA-DRB1 alleles were first divided into two groups according to the presence or absence of the RAA sequence at positions 72–74 and were denoted S and X alleles, respectively. The S alleles were subsequently divided into four groups according to the amino acid at position 71: an alanine (A), a glutamic acid (E), a lysine (K), or an arginine (R). Different groups were thus defined in the new classification: S1 for ARAA and ERAA, S2 for KRAA, S3 for RRAA, and X for all non-RAA patterns. Since an aspartic acid (D) at position 70 was reported to be protective against RA susceptibility in comparison with a glutamine (Q) or an arginine (R) at the same position [39], two additional groups were defined: S3D for DRRAA, and S3P for QRRAA or RRRAA [35,36] (Table 1).

**Table 1:** HLA–DRB1 amino acid sequence for alleles observed among rheumatoid arthritis patients and their classification according to du Montcel and colleagues

HLA-DRB1 allele	Amino acid position								Classification of du Montcel and colleagues
		
	69	70	71	72	73	74	75	76	
HLA-DRB1*0101	E	**Q**	**R**	**R**	**A**	**A**	V	D	**S3P**
HLA-DRB1*0102	-	**Q**	**R**	**R**	**A**	**A**	-	-	**S3P**
HLA-DRB1*0103	-	D	**E**	**R**	**A**	**A**	-	-	**S1**
HLA-DRB1*03	-	-	K	-	G	R	-	-	**X**
HLA-DRB1*0401	-	Q	**K**	**R**	**A**	**A**	-	-	**S2**
HLA-DRB1*0402	-	D	**E**	**R**	**A**	**A**	-	-	**S1**
HLA-DRB1*0403	-	-	-	-	-	E	-	-	**X**
HLA-DRB1*0404	-	**Q**	**R**	**R**	**A**	**A**	-	-	**S3P**
HLA-DRB1*0405	-	**Q**	**R**	**R**	**A**	**A**	-	-	**S3P**
HLA-DRB1*0407	-	-	-	-	-	**E**	-	-	**X**
HLA-DRB1*0408	-	**Q**	**R**	**R**	**A**	**A**	-	-	**S3P**
HLA-DRB1*0411	-	-	-	-	-	E	-	-	**X**
HLA-DRB1*07	-	D	-	-	G	Q	-	-	**X**
HLA-DRB1*08	-	D	-	-	-	L	-	-	**X**
HLA-DRB1*0901	-	R	-	-	-	E	-	-	**X**
HLA-DRB1*1001	-	**Q**	**R**	**R**	**A**	**A**	-	-	**S3P**
HLA-DRB1*1101	-	**D**	**R**	**R**	**A**	**A**	-	-	**S3D**
HLA-DRB1*1102	-	D	**E**	**R**	**A**	**A**	-	-	**S1**
HLA-DRB1*1103	-	D	**E**	**R**	**A**	**A**	-	-	**S1**
HLA-DRB1*1104	-	**D**	**R**	**R**	**A**	**A**	-	-	**S3D**
HLA-DRB1*12	-	**D**	**R**	**R**	**A**	**A**	-	-	**S3D**
HLA-DRB1*1301	-	D	**E**	**R**	**A**	**A**	-	-	**S1**
HLA-DRB1*1302	-	D	**E**	**R**	**A**	**A**	-	-	**S1**
HLA-DRB1*1303	-	D	**K**	**R**	**A**	**A**	-	-	**S2**
HLA-DRB1*1323	-	D	**E**	**R**	**A**	**A**	-	-	**S1**
HLA-DRB1*1401	-	R	-	-	-	E	-	-	**X**
HLA-DRB1*1402	-	**Q**	**R**	**R**	**A**	**A**	-	-	**S3P**
HLA-DRB1*1404	-	R	-	-	-	E	-	-	**X**
HLA-DRB1*15	-	Q	**A**	**R**	**A**	**A**	-	-	**S1**
HLA-DRB1*16	-	**D**	**R**	**R**	**A**	**A**	-	-	**S3D**

In the du Montcel and colleagues classification [35], the HLA–DRB1 alleles were first divided into two groups according to the presence or absence of the RAA sequence at positions 72–74, which denote S and X alleles (respectively shared epitope and nonshared epitope alleles). The S alleles were subsequently divided into four groups according to the two first amino acids at positions 70 and 71 (boldface): **S1 **for **A**RAA and **E**RAA, **S2 **for **K**RAA, **S3 **for **R**RAA (divided into **S3P **for **QR**RAA and **S3D **for **DR**RAA according to position 70), and X for all non-RAA motifs. The conventional classification of the amino acids was used, here divided into three biochemical subgroups, as follows: group 1 = G for glycine, A for alanine, V for valine, L for leucine (aliphatic amino acids (nonpolar hydrophobic)); group 2 = K for lysine, R for arginine (basic amino acids (polar and positively charged)); group 3 = E for glutamic acid, Q for glutamine (the amide corresponding to E), D for aspartic acid, and N for asparagine (the amide corresponding to D) (acidic amino acids and corresponding amides are very hydrophilic; acidic amino acids are polar and negatively charged at physiologic pH, amides are polar and uncharged, and not ionizable) [36].

### Statistical analysis

Agreements with Hardy–Weinberg equilibrium were tested using Pearson's chi-square test and Fischer's exact test when relevant. The association between the HLA-DRB1 gene polymorphism and RF or ACPAs was tested by comparing (by the chi-square test or the exact Fisher test when relevant) the distribution of positive or negative patients for RF or anti-CCP2 antibodies or AhFibA among carriers and noncarriers for each of the four groups of HLA-DRB1 alleles encoding the SE, defined according to the new classification of HLA-DRB1 alleles (S1D, S2D, S3P, S3D). Odds and odds ratios (95% confidence intervals) were also calculated. The dose effect was investigated for alleles positively or negatively associated with immunological markers using tests for the trend of the log odds.

Statistical analyses were performed using Stata Statistical Software (release 9.1 SE; Stata Corporation, College Station, TX, USA). All *P *values were two-sided, and *P *< 0.05 was considered statistically significant after correcting when relevant for multiple testing according to the Benjamini–Yekutieli 2001 method.

## Results

### Demographic and immunologic characteristics of RA patients

The main baseline demographic and immunologic characteristics of the 160 patients with early RA included in the present study were the following: 120 women (75%) and 40 men (25%); mean (± standard deviation) age, 50.31 (± 14.03) years; mean (± standard deviation) disease duration, 0.55 (± 0.02) years; number (%) RF-positive, 110/160 (68.75%); number (%) anti-CCP2 antibody-positive, 110/160 (68.75%); and number (%) AhFibA-positive, 108/160 (67.25%).

### Allele frequencies for HLA-DRB1 polymorphisms

The frequencies of HLA-DRB1 alleles, classified into five groups according to the new classification, were as follows: S1, 59/320 (18.4%); S2, 65/320 (20.3%); S3D, 42/320 (13.3%); S3P, 89/320 (27.81%); and X, 65/320 (20.31). No departures from Hardy–Weinberg equilibrium were found for HLA-DRB1 alleles classified into the five groups (*P *= 0.7171; 10 degrees of freedom).

### Relationship between HLA-DRB1 allele carrier status and RF status

Table 2 presents the status for RF among patients carrying the different HLA-DRB1 alleles encoding the SE classified into four groups according to the new classification. On the one hand, S2 carriers had a higher frequency of RF in comparison with noncarriers (odds ratio > 1 and *P *< 0.05). On the other hand, S3D and S1 carriers had a lower frequency of RF in comparison with noncarriers (odds ratio < 1 and *P *< 0.05).

**Table 2:** Relationship between HLA-DRB1 allele carrier status and rheumatoid factor status in French patients with early rheumatoid arthritis

	Carrier status		Odds ratio (95% confidence interval)	*P*	*P *for trend
				
	Yes	No			
S1 carrier					
Rheumatoid factor-positive	31 (55.4)	79 (75.9)	0.39 (0.19–0.83)	0.0118	0.0051*
Rheumatoid factor-negative	25 (44.6)	25 (24.0)			
S2 carrier					
Rheumatoid factor-positive	49 (83.1)	61 (60.4)	3.21 (1.39–7.9)	0.0028*	0.0049*
Rheumatoid factor-negative	10 (16.9)	40 (39.6)			
S3P carrier					
Rheumatoid factor-positive	57 (74.0)	53 (63.9)	1.61 (0.78–3.38)	0.1766	0.4478
Rheumatoid factor-negative	20 (26.0)	30 (36.1)			
S3D carrier					
Rheumatoid factor-positive	19 (51.4)	91 (74.0)	0.37 (0.16–0.86)	0.0145	0.0209
Rheumatoid factor-negative	18 (48.6)	32 (26.0)			

Data presented as *n *(%). Status for rheumatoid factor among 160 patients with early rheumatoid arthritis, carrying the different HLA-DRB1 alleles encoding the shared epitope classified into four groups according to the new classification. Odds ratios, 95% alpha-risk confidence interval and *P *value for exact Fisher test. The dose effect was investigated for alleles positively or negatively associated with immunological markers using tests for trend of the log odds. *Significant after correcting for multiple testing according to the Benjamini–Yekutieli 2001 method at an overall critical *P *value of 5%.

These results support the hypothesis of an association between HLA-DRB1 gene polymorphisms and RF, and the results point out the interest of the new classification of HLA-DRB1 alleles in order to differentiate predisposing and protective alleles for RF production in early RA.

### Relationship between HLA-DRB1 allele carrier status and anticitrullinated protein/peptide autoantibody status

Table 3 presents the status for anti-CCP2 antibodies or AhFibA among patients carrying the different HLA-DRB1 alleles encoding the SE classified into four groups according to the new classification. On the one hand, S2 and S3P carriers had a higher frequency of anti-CCP2 antibodies or AhFibA in comparison with noncarriers (odds ratio > 1 and *P *< 0.05). On the other hand, S3D and S1 carriers had a lower frequency of anti-CCP2 antibodies or AhFibA in comparison with noncarriers (odds ratio < 1 and *P *< 0.05).

**Table 3:** Relationship between HLA-DRB1 allele carrier status and anticitrullinated protein/peptide autoantibody status in French patients with early rheumatoid arthritis

	Carrier status		Odds ratio (95% confidence interval)	*P*	*P *for trend
				
	Yes	No			
S1 carrier					
CCP2-positive	32 (57.1)	78 (75.0)	0.44 (0.21–0.94)	0.0312	0.0134
CCP2-negative	24 (42.9)	26 (25.0)			
AhFibA-positive	31 (55.4)	77 (74.0)	0.43 (0.21–0.91)	0.0213	0.0113
AhFibA-negative	25 (44.6)	27 (26.0)			
					
CCP2-positive					
CCP2-negative	49 (83.1)	61 (60.4)	3.21 (1.39–7.9)	0.0028*	0.0049*
AhFibA-positive	10 (16.9)	40 (39.6)			
AhFibA-negative	50 (84.8)	58 (57.4)	4.12 (1.75–10.49)	0.0004*	0.0003*
CCP2-positive	9 (15.2)	43 (42.6)			
					
S3P carrier					
CCP2-positive	61 (79.2)	49 (59.0)	2.65 (1.24–5.74)	0.0066*	0.0014*
CCP2-negative	16 (20.8)	34 (41.0)			
AhFibA-positive	61 (79.2)	47 (56.6)	2.92 (1.38–6.32)	0.0025*	0.0035*
AhFibA-negative	16 (20.8)	36 (43.4)			
					
S3D carrier					
CCP2-positive	17 (45.9)	93 (75.6)	0.27 (0.12–0.63)	0.0011*	0.0009*
CCP2-negative	20 (54.1)	30 (24.4)			
AhFibA-positive	19 (51.4)	89 (72.4)	0.4 (0.18–0.93)	0.0266	0.0145
AhFibA-negative	18 (48.6)	34 (27.6)			

Data presented as *n *(%). Status for anticyclic citrullinated peptides (anti-CCP2) antibodies or antideiminated human fibrinogen autoantibodies (AhFibA) among 160 patients with early rheumatoid arthritis, carrying the different HLA-DRB1 alleles encoding the shared epitope classified into four groups according to the new classification. Odds ratios, 95% alpha-risk confidence interval and *P *value for exact Fisher test. The dose effect was investigated for alleles positively or negatively associated with immunological markers using tests for trend of the log odds. *Significant after correcting for multiple testing according to the Benjamini–Yekutieli 2001 method at an overall critical *P *value of 5%.

The interest of the new classification is that both predisposing and protective alleles for the production of ACPA are found. The effects remain significant after correction for multiple testing using the Benjamini–Yekutieli 2001 procedure implemented in STATA 9.0 (State Corporation), which corrects for an overall false discovery rate (5% here) (see Table 3). In the present analysis based on carrier status, a potential bias may be introduced by the presence of an adverse effect allele in the control group. In the analysis of the S2 effect, for example, the association may be overestimated by the presence of S3D carriers in the control group (noncarrier of S2). The effect of S2 may similarly be underestimated by the presence of S3P carriers. After controlling for the adverse effect of S3D and S1 in the analysis of S2, the association with the positivity of Ahfiba remains significant (*P *< 0.05). After controlling for the adverse effect of S2 and S3P in the analysis of S3D, the association with negativity of anti-CCP2 remained significant.

These results support the hypothesis of an association between HLA-DRB1 gene polymorphisms and ACPAs, and point out the interest of the new classification of HLA-DRB1 alleles in order to differentiate predisposing and protective alleles for ACPA production in early RA.

## Discussion

The results of the present study confirm previous evidence of an association between HLA-DRB1 gene polymorphisms and RF or ACPAs in RA. Furthermore, the results point out the interest of the new classification of HLA-DRB1 alleles in order to differentiate predisposing and protective alleles for autoantibody production in early RA.

The results of the present study confirm previous evidence of an association between HLA-DRB1 gene polymorphisms and autoantibody production in RA. We found a positive association between carriers of HLA-DRB1*SE^+ ^alleles (HLA-DRB1*0401, HLA-DRB1*0404, HLA-DRB1*0405, HLA-DRB1*0408, HLA-DRB1*1001) and RF or ACPA production, while we did not find any negative association between carriers of HLADRB1*SE^- ^alleles and RF or ACPA production (data not shown). An association between HLA-DRB1*04 or HLA-DRB1*SE^+ ^alleles and RF has been reported in some studies [12,30,40] but rejected in others [12,32,41]. An association between HLA-DRB1*01, HLA-DRB1*04 or HLA-DRB1*SE^+ ^alleles and ACPAs was more constantly reported in European or North American RA patients [9,12,24,30,32,40,42-45]. Since the presence of RF was strongly correlated with that of ACPAs in most of these studies, several groups investigated whether these associations between HLA-DRB1 gene polymorphisms and RF or ACPAs were independent. These studies showed that the association between HLA-DRB1*SE^+ ^alleles and ACPAs is constantly stronger than the association between HLA-DRB1*SE^+ ^alleles and RF. Furthermore, they suggested that the association between HLA-DRB1*SE^+ ^alleles and ACPAs is independent of the RF status, leading to the conclusion that HLA-DRB1*SE^+ ^alleles are primarily associated with the presence of ACPAs, but not with the presence of RF [24,32,41].

The results of the present study indicate the interest of the new classification of HLA-DRB1 alleles to differentiate predisposing and protective alleles for autoantibody production in early RA. This new classification, which is based on an initial split of HLA-DRB1 alleles into two groups according to the presence (S alleles) or absence (X alleles) of the RAA sequence at positions 72–74, subsequently divides S alleles into four groups according to the amino acids at positions 71 and 70. Most of the previous studies, based on the common classification, identified HLA-DRB1*101, HLA-DRB1*0401, HLA-DRB1*404 and HLA-DRB1*1001 as predisposing alleles for ACPA production in RA, with a significant dose effect in patients carrying two of these predisposing alleles [9,12,32,44]. Only a few association studies reported an HLA-DRB1 allelic protective effect for ACPA production in RA. In these studies, HLA-DRB1*03 was associated with ACPA-negative RA and decreased titers of ACPAs, even in the presence of an SE allele [32,45]. In the new classification of HLA-DB1 allelles, HLA-DRB1*03 is not taken into account separately since it is classified into the X group of alleles, which do not encode the SE sequence. In the present study, complementary analysis did not show any association between HLA-DRB1*03 carrier status and RF or ACPA production (data not shown). The use of the classification by du Montcel and colleagues suggests a risk hierarchy in ACPA production in early RA patients: the S2 (KRAA at positions 71–74) and S3P (QRRAA or RRRAA at positions 70–74) alleles conferring predisposition, while the S1 (ARAA or ERAA at positions 71–74) and S3D (DRRAA at positions 70–74) alleles confer protection, in comparison with X (non-RAA patterns at positions 72–74).

The use of the new classification of HLA-DRB1 alleles proposed by du Montcel and colleagues seems to provide different pictures of the relative contribution of the HLA-DRB1 locus to RA pathogenesis. This relative contribution is not restricted to ACPA production, but also includes risk hierarchy for RA susceptibility and structural severity [35-37].

Trying to understand the findings of genetic association/linkage studies in complex multifactorial diseases, such as RA, in light of the amino acid alignment of a protein encoded by a candidate gene remains a challenging task. In fact, the interactions between HLA-DRB1 molecules and citrullinated peptides may impact RA pathogenesis in several ways. For example, a previous study conducted in DR4-IE transgenic mice demonstrated that the conversion of arginine to citrulline at the peptide side-chain position interacting with the SE significantly increases peptide–MHC affinity and leads to the activation of CD4^+ ^T cells, suggesting that HLA-DRB1 alleles encoding the SE could initiate an autoimmune response to citrullinated self-antigens [46].

## Conclusion

Although no formal conclusions on causality can be drawn from the present association study, our findings indicate the interest of this new classification of HLA-DRB1 alleles in order to differentiate predisposing and protective alleles for autoantibody production in RA.

## Abbreviations

ACPA = anticitrullinated protein/peptide autoantibody; AhFibA = antideiminated human fibrinogen autoantibodies; anti-CCP = anticyclic citrullinated peptide; ELISA = enzyme-linked immunosorbent assay; HLA = human leukocyte antigen; MHC = minor histocompatibility complex, PCR = polymerase chain reaction; RA = rheumatoid arthritis; RF = rheumatoid factor; SE = shared epitope.

## Competing interests

The authors declare that they have no competing interests.

## Authors' contributions

P-AG and ACo took the leadership of the study in both clinical immunological and statistical aspects. FC and PD contributed specifically to the genotyping. GS and LN were specifically in charge of the autoantibody study. AC-T contributed to the statistical analysis. BM, ACa and J-FB contributed through the assessment of the RMP cohort.
